# Risk factors and nomogram development for lymph node metastasis in early-onset early-stage gastric cancer: a retrospective cohort study

**DOI:** 10.3389/fonc.2025.1544758

**Published:** 2025-04-30

**Authors:** Binghe Zhao, Mingyu Gu, Zijian Wang, Jie Li, Minghai Wen, Di Wu, Shuo Li, Lu Liu, Xinxin Wang

**Affiliations:** ^1^ Department of General Surgery, The First Medical Center of Chinese PLA General Hospital, Beijing, China; ^2^ Medical College, Nankai University, Tianjin, China

**Keywords:** early onset gastric cancer, early onset early stage gastric cancer, lymph node metastasis, nomogram, endoscopic submucosal dissection

## Abstract

**Background:**

The incidence of early onset gastric cancer(EOGC) is increasing. However, few studies have focused on early onset early stage gastric cancer(EEGC). The aim of this study was to determine the threshold age of patients with EOGC, identify the clinicopathological characteristics associated with lymph node metastasis(LNM) in EEGC, and develop a predictive model for LNM in EEGC.

**Methods:**

A retrospective cohort study was conducted, including 1765 patients with early-stage gastric cancer. Logistic inflection point and stratified analysis were used to determine the threshold age. 266 patients met the criteria for EEGC and were included for further analysis. The patients were divided into two groups for the purposes of the study: a training dataset and an external validation dataset. The division of patients into these two groups was conducted in accordance with the time of surgery, with the ratio of patients in each group being approximately 7:3.Univariate and multivariate logistic regression analysis were used to identify LNM risk factors. A predictive nomogram was developed and validated using calibration plots and the area under the curve (AUC).The constructed logistic regression model was then validated using the external validation dataset.

**Results:**

The threshold age for EOGC was determined to be 45 years. Of the 266 patients with EEGC, 20.7% had LNM. Tumor maximum diameter and lymphovascular invasion were identified as independent risk factors for LNM. The nomogram demonstrated high predictive accuracy, with an AUC of 0.809.

**Conclusions:**

This study demonstrated that tumor maximum diameter and lymphovascular invasion were independent risk factors for LNM in EEGC. The predictive nomogram showed promising accuracy and might assist in identifying patients at higher risk of LNM, potentially informing treatment strategies. Given the relatively high LNM rate, endoscopic submucosal dissection may not be suitable for EEGC patients. Further large-scale multicenter studies are needed to deepen the understanding of this population and to confirm these findings.

## 1 Introduction

Early-onset gastric cancer (EOGC) refers to gastric cancer that occurs at a younger age. Currently, there is no universally defined onset age. Some studies used a threshold of 40 or 50 years old (1–4), but the academic community at large considers the threshold age for EOGC to be 45 years (5, 6). The incidence of early onset gastric cancer is increasing (7). Numerous studies have highlighted the distinct characteristics of EOGC compared to late-onset cases, emphasizing the significance of recognizing it as a separate pathological entity (8)^-^ (9). Several studies showed that EOGC is more aggressive than traditional gastric cancer (10).

Early stage GC is limited to mucosal (T1a) or submucosal (T1b), regardless of lymph node metastasis (LNM) (11). According to the sixth edition of the Japanese Gastric Cancer Treatment Guidelines, patients with early gastric cancer may be eligible for endoscopic submucosal dissection (ESD) in addition to radical gastrectomy (12). ESD is particularly indicated for tumors with a minimal likelihood of LNM and that are amenable to an bloc resection (13, 14),which prioritizes organ preservation and quality of life (15). Previous studies have suggested that EOGC tends to exhibit lower differentiation and more advanced staging at diagnosed compared to the older patients (16, 17). Consequently, EEGC might be more prone to LNM due to the more aggressive biological behaviour and advanced pathological features observed in EOGC. Therefore, understanding the incidence and risk factors of LNM in EEGC is crucial for guiding treatment strategies. However, to date few studies focus on EEGC patients. Further investigation is needed to better define the optimal management strategies for EEGC, especially in terms of LNM risk and therapeutic approaches.

Consequently, we conducted a retrospective analysis aimed at exploring the appropriate definition of EOGC and the risk factors associated with LNM in EEGC. Furthermore, we attempted to construct a nomogram to predict the occurrence of LNM to identify the appropriate candidates for ESD among these patients.

## 2 Materials and methods

### 2.1 Patients

**Figure 1:**
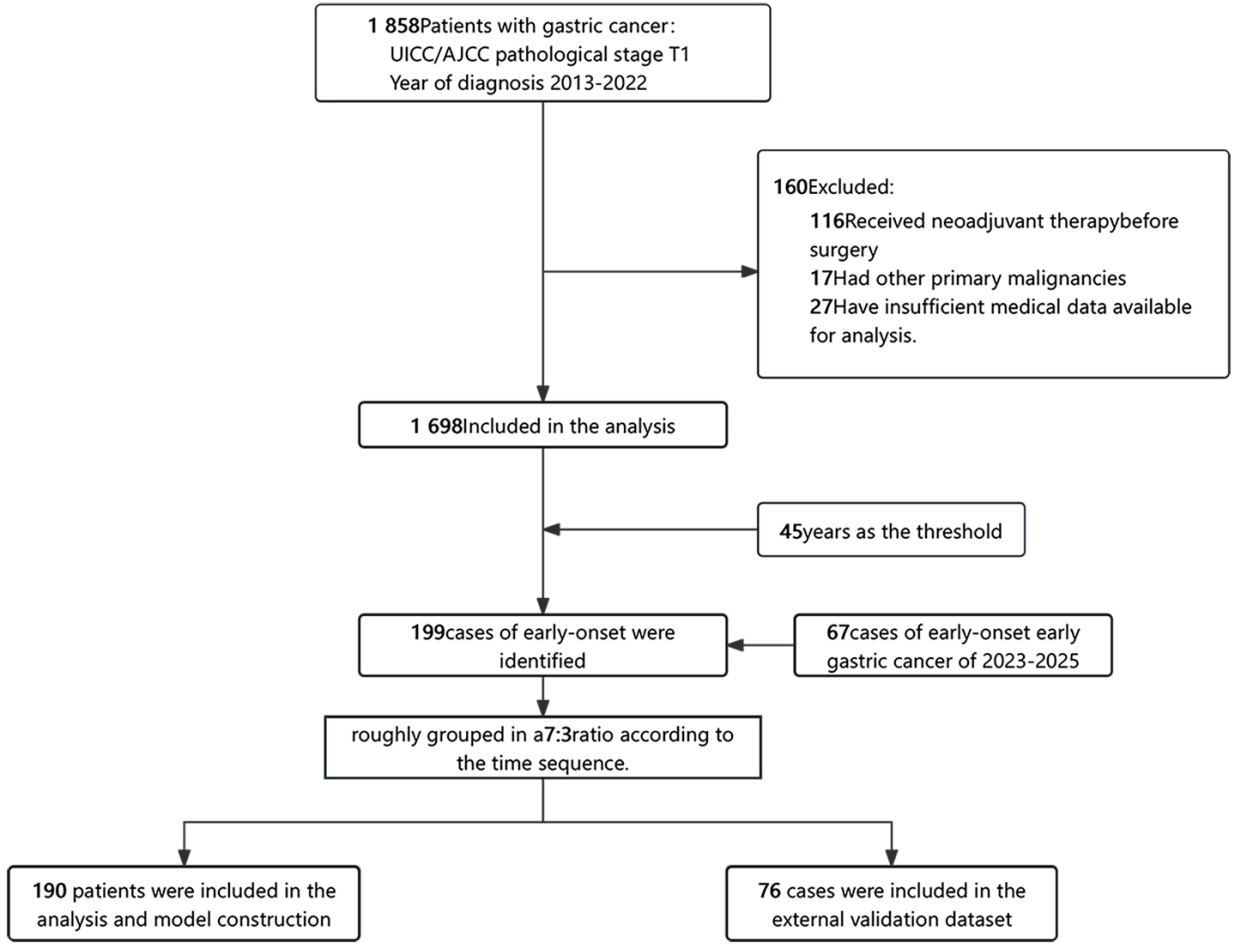
Flowchart of participant selection.

This study included patients who underwent a radical resection for primary gastric cancer and were histologically proven to have pT1 gastric adenocarcinoma, as defined by the 8th edition of the AJCC TNM staging system, at the First Medical Center of the PLA General Hospital in Beijing, China, from 2013 to 2025. All patients were of Asian ethnicity. For inclusion in this analysis, patients had to meet the following criteria: (1) they were diagnosed with pT1 stage gastric cancer based on histological examination; (2) they possessed comprehensive medical information relevant to the study; (3) they underwent surgery without any neoadjuvant therapy; (4) they had no other concurrent malignant tumors. In total. This study was approved by the Ethics Committee of Chinese PLA General Hospital. (No. S2023-275-01). Due to the study’s retrospective design, the requirement for informed consent was waived. Finally, a total of 1,765 patients were included in the study, of whom 266 had early-stage gastric cancer (**Figure 1**).

### 2.2 Patient characteristics and clinical data

This article analyzed various clinical characteristics, including age, gender, family history of gastric cancer, body mass index (BMI), and pathological characteristics. The pathological characteristics considered for analysis included tumor location, tumor maximum diameter, invasion depth, histological type, lymphovascular invasion, perineural invasion and lymph node status. Additionally, laboratory indicators such as Serum Markers (CEA, AFP, CA19-9, CA15-3, CA125, CA724) and immune cell indicators (neutrophils, lymphocytes, monocytes) were included. The histological types were reviewed and categorized into four groups: adenocarcinoma, signet ring cell adenocarcinoma, mixed adenocarcinoma with signet ring cell carcinoma and mixed adenocarcinoma with neuroendocrine carcinoma. Two independent and experienced pathologists reviewed hematoxylin-eosin (H&E)-stained slides from each case, and in instances of inconsistent diagnoses between the two pathologists, a third pathologist was consulted.

### 2.3 Statistical analysis

Categorical variables were compared using Chi-square or Fisher’s exact test, and continuous variables were compared using analysis of variance (ANOVA) or Kruskal-Wallis test. Statistical analyses were performed using the R software package (http://www.R-project.org, The R Foundation) and Free Statistics software version 1.8. Two-tailed tests were performed, and a significance level of P value < 0.05 was used to determine statistical significance.

## 3 Results

### 3.1 Optimal age threshold for EOGC

**Figure 2:**
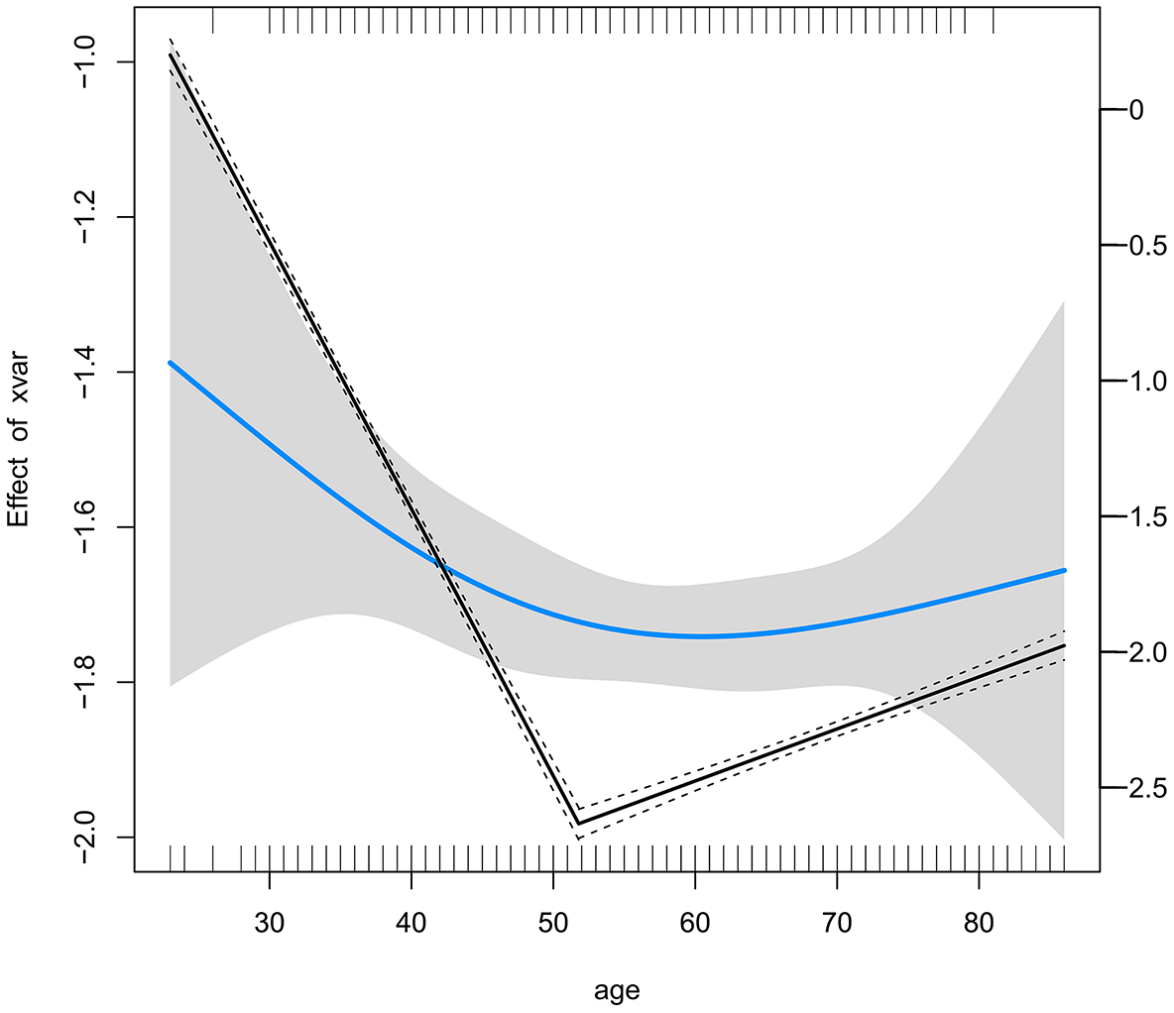
Threshold Effect of Age on Lymph Node Metastasis Risk Identified by Logistic Inflection Point Analysis.

A logistic inflection point analysis was conducted to evaluate the relationship between age and the probability of LNM in the patients from 2013 to 2022. The analysis identified a significant turning point at 51.8 years (95% CI: 51.1–52.5), indicating a distinct change in the effect of age on the probability of LNM. Prior to the turning point, the slope of the relationship between age and LNM was significantly negative (OR: 0.957, 95% CI: 0.918–0.997, *P* = 0.035), indicating a decreasing probability of LNM with increasing age. However, beyond the turning point, the slope flattened and became statistically nonsignificant (OR: 0.997, 95% CI: 0.974–1.020, *P* = 0.769), indicating a plateau in the effect of age on LNM probability in older patients (**Figure 2**).

To further determine the optimal age threshold for defining EOGC, we conducted a stratified analysis of patients aged between 40 and 51 years. For each age cutoff (e.g., ≤40 vs. >40, ≤41 vs. >41), a contingency table was constructed to compare the LNM rates between the two groups. The results demonstrated that cutoffs at 42 years (OR: 1.72, 95% CI: 1.09–2.71, *P* = 0.027), 43 years (OR: 1.59, 95% CI: 1.03–2.47, *P* = 0.048), 45 years (OR: 1.54, 95% CI: 1.04–2.19, *P* = 0.036), and 46 years (OR: 1.51, 95% CI: 1.04–2.19, *P* = 0.036) showed statistical significance (**Supplementary Table S1**). The results indicated that defining EOGC at 46 years is also reasonable and practical. However, to align with the widely accepted definition within the academic community, our study adopted 45 years as the threshold to ensure consistency and comparability with existing research, and included 199 cases of early-onset gastric cancer.

### 3.2 Clinicopathological characteristics

Among the 190 cases of EEGC patients of training dataset, the age of the patients ranged from 24 to 45 years, with a mean age of 39 ± 5 years. The median tumor size was 1.8 cm. Among these cases, 37 patients exhibited LNM, resulting in a metastasis rate of 19.5%. The influence of clinical factors—including age, sex, BMI, tumor maximum diameter, location, tumor marker levels, and pathological characteristics—on LNM was assessed. Patients with LNM tended to have a larger median tumor maximum diameter (2.0 cm vs. 1.5 cm, *P* = 0.001) and a significantly higher incidence of lymphovascular invasion (27.0% vs. 5.2%, *P* < 0.001) compared to those without metastasis. Submucosal invasion was also more common in the LNM+ group (62.2% vs. 34.6%, *P* = 0.002). No significant differences were observed between the two groups for gender, age, family history, BMI, tumor markers, or neural invasion. The demographic and clinicopathological data for both cohorts are summarized in **Table 1**.

**Table 1:** Characteristics of early onset early stage gastric cancer with and without lymph node metastasis.

Characteristic	Patients No.(%)			*P* value
	Total	LMN- (n = 153)	LMN+(n = 37)	
Gender, n (%)				0.367
Female	85 (44.7)	66 (43.1)	19 (51.4)	
Male	105 (55.3)	87 (56.9)	18 (48.6)	
Age, Mean ± SD	39.09 ± 4.99	39.24 ± 4.90	38.46 ± 5.37	0.394
Family history, n (%)				0.421
No	165 (86.8)	131 (85.6)	34 (91.9)	
Yes	25 (13.2)	22 (14.4)	3 (8.1)	
Neutrophils, Mean ± SD	0.55 ± 0.08	0.55 ± 0.08	0.57 ± 0.09	0.111
Lymphocyte, Mean ± SD	0.35 ± 0.08	0.36 ± 0.07	0.33 ± 0.08	0.094
Monocyte, Mean ± SD	0.07 ± 0.02	0.07 ± 0.02	0.07 ± 0.02	0.394
Smoke, n (%)				0.09
No	138 (72.6)	107 (69.9)	31 (83.8)	
Yes	52 (27.4)	46 (30.1)	6 (16.2)	
Drink, n (%)				0.392
No	122 (64.2)	96 (62.7)	26 (70.3)	
Yes	68 (35.8)	57 (37.3)	11 (29.7)	
BMI, Mean ± SD	23.97 ± 4.08	23.99 ± 4.16	23.90 ± 3.76	0.906
Location, n (%)				0.829
Gastric angle	40 (21.1)	33 (21.6)	7 (18.9)	
Gastric antrum	91 (47.9)	71 (46.4)	20 (54.1)	
Gastric corpus	58 (30.5)	48 (31.4)	10 (27)	
Gastroesophageal junction	1 (0.5)	1 (0.7)	0 (0)	
Degree of differentiation, n (%)				0.28
High	8 (4.2)	8 (5.2)	0 (0)	
Low	134 (70.5)	109 (71.2)	25 (67.6)	
Middle	48 (25.3)	36 (23.5)	12 (32.4)	
Pathological type, n (%)				0.021
Adenocarcinoma	47 (24.7)	37 (24.2)	10 (27)	
Adenocarcinoma with Neuroendocrine	2 (1.1)	1 (0.7)	1 (2.7)	
Adenocarcinoma with Signet-ring cell	117 (61.6)	91 (59.5)	26 (70.3)	
Signet-ring cell	24 (12.6)	24 (15.7)	0 (0)	
Invasion depth, n (%)				0.002
Mucosa	114 (60.0)	100 (65.4)	14 (37.8)	
Submucosa	76 (40.0)	53 (34.6)	23 (62.2)	
Lymphovascular space invasion, n (%)				< 0.001
No	172 (90.5)	145 (94.8)	27 (73)	
Yes	18 (9.5)	8 (5.2)	10 (27)	
Neural invasion, n (%)				1
No	187 (98.4)	150 (98)	37 (100)	
Yes	3 (1.6)	3 (2)	0 (0)	
Tumor Markers				
CEA, Median (IQR)	1.4 (0.9, 2.0)	1.4 (0.9, 1.9)	1.5 (0.9, 2.7)	0.465
AFP, Median (IQR)	2.5 (1.7, 3.4)	2.4 (1.7, 3.4)	2.6 (1.8, 3.0)	0.792
CA125, Median (IQR)	9.3 (7.0, 13.6)	9.2 (7.0, 13.2)	10.4 (7.6, 15.1)	0.285
CA19.9, Median (IQR)	8.4 (5.5, 13.1)	8.1 (5.7, 13.3)	9.2 (5.4, 12.5)	0.65
CA15.3, Median (IQR)	6.6 (4.9, 8.4)	6.7 (5.1, 8.4)	6.0 (4.8, 9.3)	0.66
CA724, Median (IQR)	1.4 (1.0, 2.6)	1.4 (1.0, 2.7)	1.5 (1.0, 2.4)	0.87
Tumor maximum diameter, Median (IQR)	1.8 (1.0, 2.5)	1.5 (1.0, 2.5)	2.0 (1.8, 3.5)	0.001

LNM+, positive lymph node metastases; LNM-, negative lymph node metastasis; BMI, body mass index; SD, standard deviation; IQR, interquartile range.

### 3.3 Risk factors of lymph node metastasis

To identify the risk factors associated with LNM, we performed univariate and multivariate logistic regression analyses in training dataset. In the univariate analysis, lymphovascular invasion (OR: 6.71; 95% CI: 2.43–18.55; *P* < 0.001), invasion depth (OR: 3.11; 95% CI: 1.47–6.52; *P* = 0.003), and maximum tumor diameter (OR: 1.66; 95% CI: 1.24–2.22; *P* = 0.001) were identified as significant risk factors for LNM. No other variables were found to be significantly associated with LNM. The results of the univariate logistic regression analysis are presented in **Table 2**. In the multivariate logistic regression analysis, lymphovascular invasion (OR: 5.28; 95% CI: 1.52–18.35; P = 0.009) and maximum tumor diameter (OR: 1.45; 95% CI: 1.06–1.99; *P* = 0.021) remained independent risk factors for LNM. The results of the multivariate logistic regression analysis are presented in **Table 3**. Subsequently, we constructed a nomogram based on the multivariate analysis results. The accuracy of this nomogram was evaluated using the Harrell C-index and the area under the curve (AUC).

**Table 2:** Univariate logistic regression analysis of risk factors for lymph node metastasis.

Variable	OR(95%CI)	*P* value
Gender(ref=Female)	0.72 (0.35~1.48)	0.368
Age	0.97 (0.9~1.04)	0.392
Family history (ref=No)	0.53 (0.15~1.86)	0.318
CEA	1.21 (0.93~1.57)	0.155
AFP	1.03 (0.97~1.1)	0.337
CA125	1.02 (0.99~1.04)	0.242
CA19.9	1 (0.96~1.05)	0.892
CA15.3	1.01 (0.91~1.12)	0.888
CA724	0.96 (0.85~1.09)	0.514
Neutrophils	36.56 (0.43~3105.86)	0.112
Lymphocyte	0.02 (0~2.06)	0.096
Monocyte	17887.08 (0~99317136583084.2)	0.392
BMI	0.99 (0.91~1.09)	0.906
Location(ref=Gastric angle)		
Gastric antrum	1.33 (0.51~3.45)	0.56
Gastric corpus	0.98 (0.34~2.84)	0.973
Gastroesophageal junction	0 (0~Inf)	0.988
Degree of differentiation(ref=High)		
Low	3589761.62 (0~Inf)	0.986
Middle	5217120.22 (0~Inf)	0.985
Pathological type (ref=Adenocarcinoma)		
Adenocarcinoma with Neuroendocrine	3.7 (0.21~64.51)	0.37
Adenocarcinoma with Signet-ring cell	1.06 (0.46~2.41)	0.895
Signet-ring cell	0 (0~Inf)	0.99
Invasion depth(ref=Mucosa)	3.1 (1.47~6.52)	0.003
Lymphovascular space invasion(ref=No)	6.71 (2.43~18.55)	<0.001
Neural invasion(ref=No)	0 (0~Inf)	0.987
Tumor maximum diameter	1.66 (1.24~2.22)	0.001

OR, odds ratio; CI, confidence interval; BMI, body mass index.

**Table 3:** Multivariate logistic regression analysis of independent risk factors for lymph node metastasis.

Variable	OR(95%CI)	*P* value
Gender(ref=Female)	0.61 (0.24~1.56)	0.3
Age	0.93 (0.86~1.01)	0.072
Family history(ref=No)	0.53 (0.13~2.09)	0.363
BMI	1.04 (0.91~1.19)	0.56
Location(ref=Gastric angle)		
Gastric antrum	1.59 (0.51~4.91)	0.423
Gastric corpus	1.05 (0.3~3.61)	0.94
Gastro esophageal junction	0 (0~Inf)	0.998
Degree of differentiation(ref=High)		
Low	20654660.03 (0~Inf)	0.994
Middle	23650055.27 (0~Inf)	0.994
Pathological type (ref=Adenocarcinoma)		
Adenocarcinoma with Neuroendocrine	0.3 (0.01~9.79)	0.499
Adenocarcinoma with Signet-ring cell	0.77 (0.24~2.4)	0.647
Signet-ring cell	0 (0~Inf)	0.989
Invasion depth(ref=Mucosa)	2.26 (0.88~5.85)	0.092
Lymphovascular space invasion(ref=No)	4.66 (1.28~16.89)	0.019
Neural invasion(ref=No)	0 (0~Inf)	0.996
Tumor maximum diameter	1.43 (1.04~1.96)	0.026

OR, odds ratio; CI, confidence interval; BMI, body mass index.

### 3.4 Development and validation of predictive models for LNM risk

**Figure 3:**
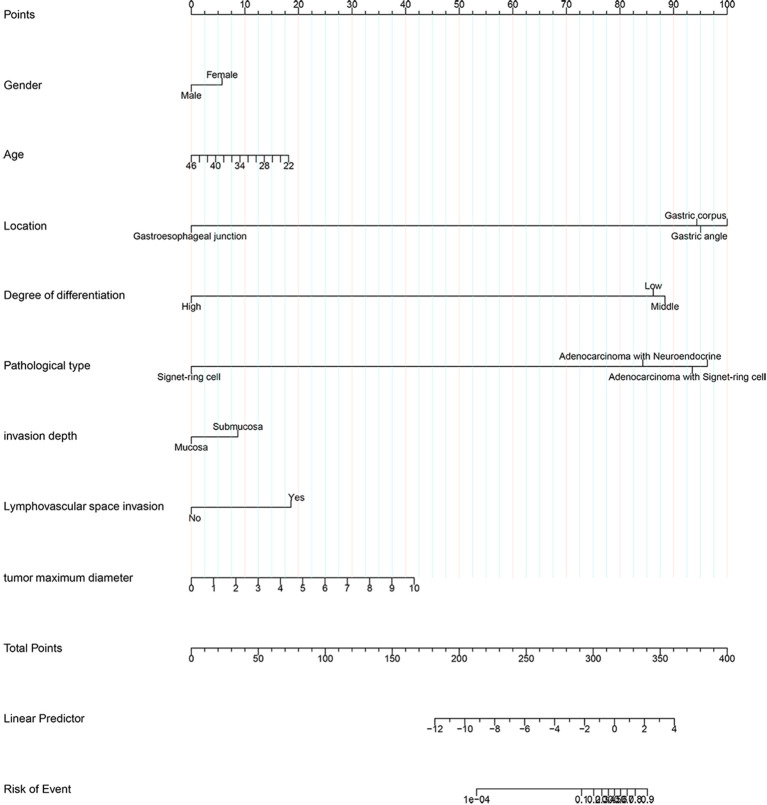
Nomogram based on clinicopathological characteristics.

**Figure 4:**
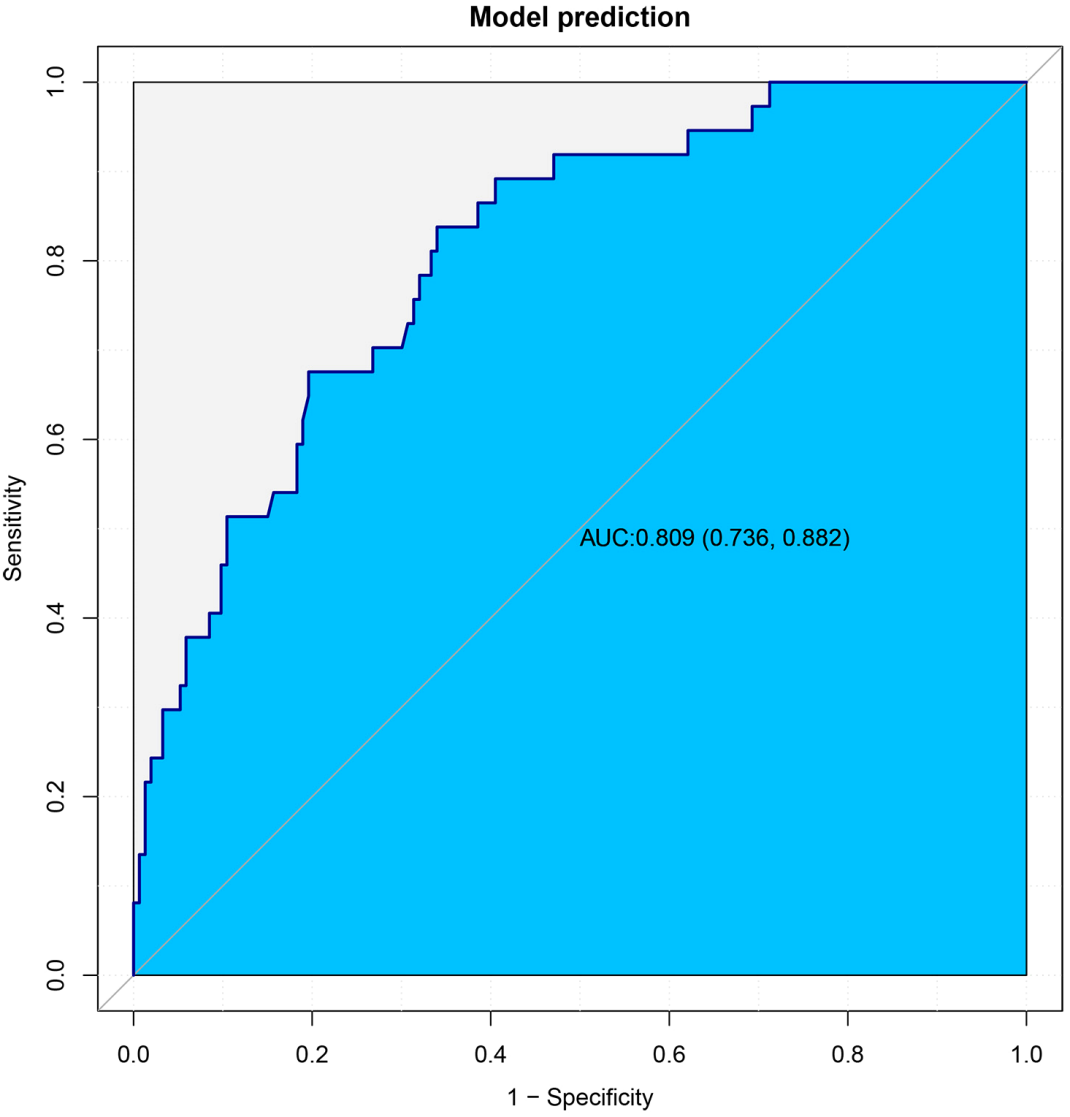
The ROC curves of the training cohort.

This article presented a predictive model and visualized it by constructing nomogram (**Figure 3**). The model incorporated clinical laboratory indicators and pathological data that can be obtained for ESD, including gender, age, tumour size, tumour location, degree of tumour differentiation, tumour pathological type, invasion depth and lymphovascular invasion. ROC subject working characteristic curves were constructed to evaluate the effectiveness of the model. The area AUC of the model was 0.809 (95%CI: 0.736-0.882), and the C index for predicting LNM was 0.739 (**Figure 4**). **Supplementary Figure S1** showed the calibration curve for predicting LNM in EEGC patients which demonstrated a strong correlation between nomogram predictions and actual outcomes.

**Figure 5:**
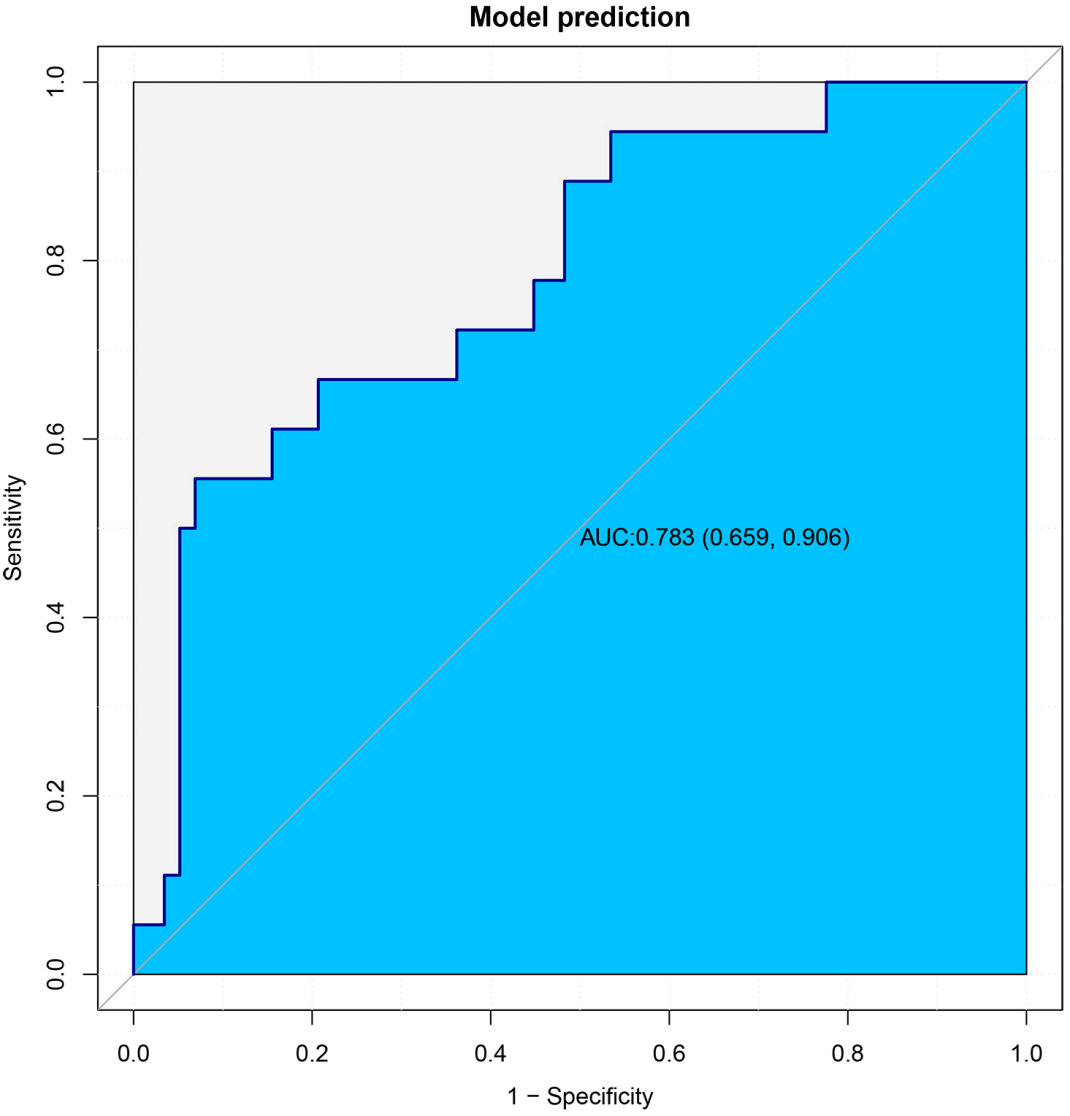
The ROC curves of the external validation cohort.

In the subsequent stage of the study, the 76 cases that had been retained were used as an external validation dataset, with the objective of validating the model that had been constructed. The demographic and clinicopathological data of the patients in the external validation dataset are summarized in **Supplementary Table S2**. The results demonstrated that the area under the curve (AUC) of the model in the external validation dataset was 0.783, which indicates that the model that was built has good discriminative ability in the external validation dataset (**Figure 5**).

## 4 Discussion

The present study included 1,765 patients with early gastric cancer. Through inflection point analysis and stratified analysis, it was determined that the definition of EOGC as <46 years was also reasonable. Furthermore, the results provided additional support for the academic definition of EOGC. In accordance with the widely accepted definition in academia, 190 patients with EOGC were included for further analysis. Notable differences in maximum tumor diameter, lymphovascular invasion and invasion depth were identified in relation to the presence of LNM in this study. Univariate analysis indicated a correlation among them, while multivariate analysis confirmed that tumor maximum diameter and lymphovascular invasion were independent risk factors for LNM in EEGC patients, consistent with previous research findings (18).

A multicenter study revealed that the incidence of LNM in patients with early gastric cancer was significantly higher among young women, particularly those with tumors located in the lower third of the stomach, greater than 2 cm in size, of the depressed type, poorly differentiated or nondifferentiated, and exhibiting lymphovascular invasion, nerve invasion, and submucosal infiltration (19). Another study demonstrated that multivariate analyses identified lymphovascular emboli, CA19–9 levels, ulceration, tumor size, tumor infiltration, and histological grade as independent risk factors for LNM (20). Additionally, factors significantly associated with LNM included age, sex, lymphatic invasion, depth of invasion, anatomical site, circumferential location, gross type, differentiation, and tumor size (21). Mixed histologic type was an independent risk factor for LNM in early gastric cancer patients (22). These findings were generally consistent with our experimental results. Discrepancies in experimental outcomes may arise from variations in the studied populations. However, there were relatively few articles exploring the factors and predictive models of LNM in patients with early-onset gastric cancer.

Our study focused on the clinical pathological characteristics and risk factors of LNM in EEGC for the first time. However, when exploring the risk factors for LNM, the correlation between differentiation degree and lymph node status appeared to be weak. Among the 37 patients with LNM from 2018 to 2022, no cases were observed in patients with upper gastric cancer. According to the absolute indications for ESD surgery (12), of the 135 patients with a tumor maximum diameter ≤ 2cm and infiltration limited to the mucosa, 20 (14.8%) exhibited LNM. The risk of LNM is relatively high compared to general early gastric cancer (14), which suggested that ESD is not suitable for EEGC (13). Studies showed that percentage of monocytes, hematocrit (HCT), and lymphocyte-monocyte ratio (LMR) might predict the metastasis of lymph node^19^. But our study showed that there was no difference between two groups. These findings indicated a need for further investigation into the assessment of tumour markers in patients prior to treatment, with a view to determining any potential impact. Preoperative diagnostic methods for LNM included endoscopic ultrasound, computed tomography (CT) and magnetic resonance imaging (MRI). However, the perigastric lymph nodes are situated within the perigastric omentum tissue in the abdominal cavity, increasing the difficulty and risk associated with imaging, localization, and puncture procedures. Endoscopic ultrasonography is effective for evaluating tumor size and depth of invasion prior to surgery (23). Among the 37 patients with LNM, 31 underwent preoperative enhanced CT scans, but only 5 cases indicated LNM (**Supplementary Table S3**). It proved that for patients with early-onset gastric cancer, enhanced CT examine may have weaker detection efficacy for LNM in early gastric cancer. Therefore, the screening of LNM remained an area deserving further investigation.

Our study highlighted that maximum tumor diameter and lymphovascular invasion were independent risk factors associated with LNM in EEGC patients. More importantly, we constructed a predictive model based on available data based on ESD to assess the risk of lymph node metastasis. The model offered a considerable clinical insight into assessing the necessity of additional surgery following ESD, providing partial data support and scientific rationale for doctor’s decision-making. While we have refined our experimental design as much as possible, our research still leaves some limitations. (1) It was a single-center retrospective analysis, which may introduce potential selection bias; (2) The considerable time span of patient enrollment could introduce variability due to advancements in diagnostic and treatment modalities for gastric cancer. Factors such as the extent of resection, the scope of intraoperative lymph node dissection, the pathological detection method, and the experience of postoperative pathologists may influence LNM detection in early gastric cancer and lead to false negatives; and (3) Some missing data were imputed using multiple imputation, which may introduce bias.

In the future, we will combine the predictive model in this article and conduct proteomic analysis on this disease to explore the biomarkers associated with LNM in patients with early-onset gastric cancer, so as to make predictions in preoperative diagnosis.

## 5 Conclusion

This study supports the existing academic consensus that 45 years is a reasonable threshold age for defining EEGC. Tumor maximum diameter and lymphovascular invasion were identified as significant risk factors for LNM in EEGC. Given the relatively high rate of LNM in this population, ESD might not be appropriate for many EEGC patients. The developed predictive nomogram showed potential to facilitate risk stratification and inform the necessity of additional surgery following ESD. Further large-scale multicenter studies are essential to validate these findings and to refine predictive models for better clinical application.

## Data Availability

The raw data supporting the conclusions of this article will be made available by the authors, without undue reservation.

## Supplementary material

Supplementary Figure 1 The calibration curve for predicting LNM in EEGC patients.
