# Supplementary figures and images for: Risk factors and nomogram development for lymph node metastasis in early-onset early-stage gastric cancer: a retrospective cohort study
Source: Front Oncol. 2025 Apr 30;15:1544758. doi: 10.3389/fonc.2025.1544758 (PMC12074922; doi:10.3389/fonc.2025.1544758)

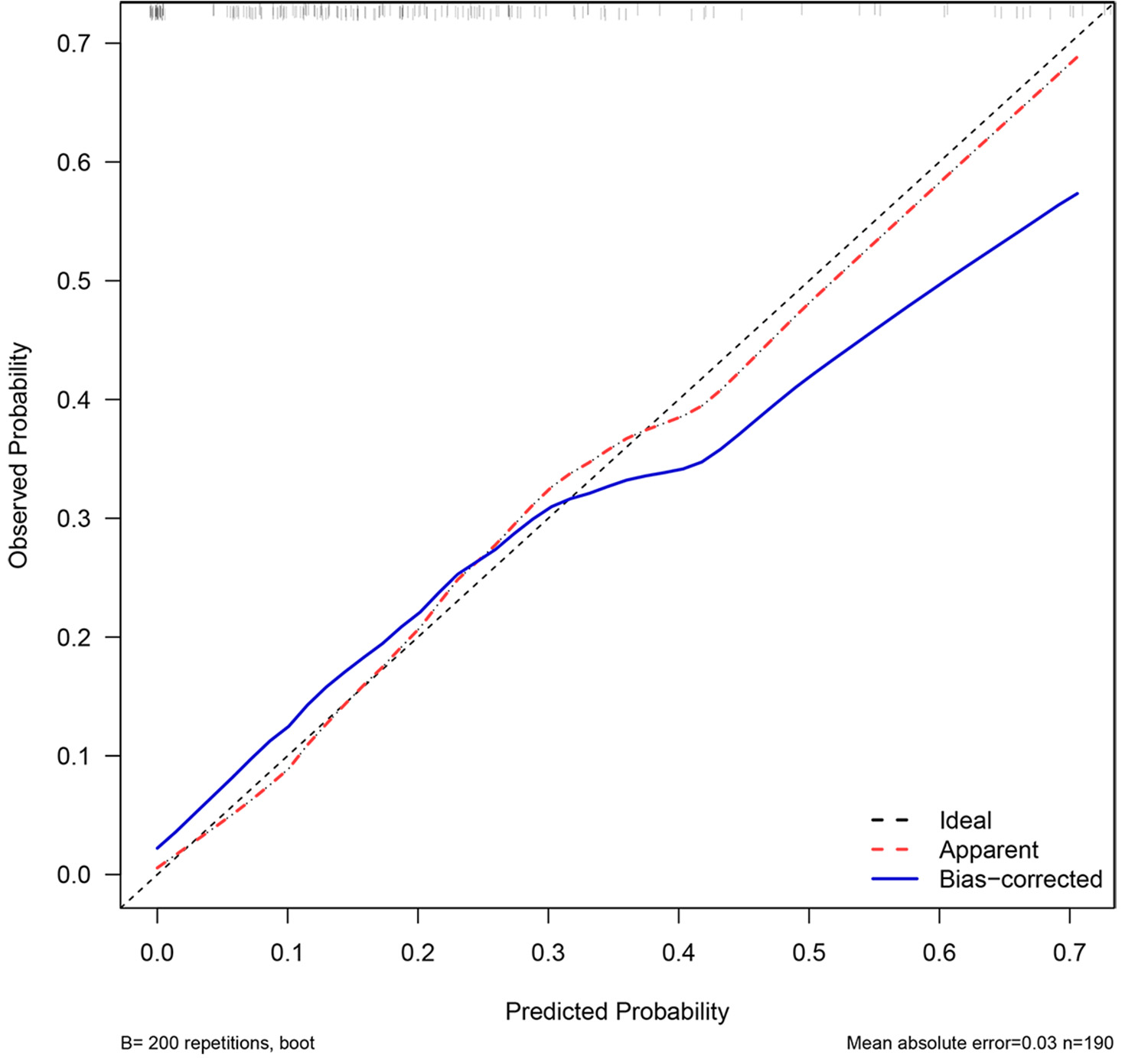

Supplement: Supplementary Figure 1 — The calibration curve for predicting LNM in EEGC patients. [file Image1.jpeg]
