# Supplementary material for: Risk factors and nomogram development for lymph node metastasis in early-onset early-stage gastric cancer: a retrospective cohort study
Source: Front Oncol. 2025 Apr 30;15:1544758. doi: 10.3389/fonc.2025.1544758 (PMC12074922; doi:10.3389/fonc.2025.1544758)
Supplement: Supplementary file 2 [file Table1.docx]

**Table S1. Stratified analysis of lymph node metastasis rate by age cutoffs**

| **Cutoff Age** | **Chi-Square** | ***P* Value** | **OR (95% CI)** |
| --- | --- | --- | --- |
| 40 | 2.48 | 0.12 | 1.56 (0.94, 2.59) |
| 41 | 3.18 | 0.07 | 1.61 (0.99, 2.62) |
| **42** | 4.91 | **0.03** | 1.72 (1.09, 2.71) |
| 43 | 3.92 | 0.05 | 1.59 (1.03, 2.47) |
| 44 | 3.68 | 0.06 | 1.53 (1.01, 2.31) |
| **45** | 4.41 | **0.04** | 1.54 (1.05, 2.28) |
| **46** | 4.39 | **0.04** | 1.51 (1.04, 2.19) |
| 47 | 3.21 | 0.07 | 1.42 (0.99, 2.04) |
| 48 | 1.27 | 0.26 | 1.25 (0.87, 1.78) |
| 49 | 0.55 | 0.46 | 1.16 (0.82, 1.63) |
| 50 | 0.52 | 0.47 | 1.14 (0.82, 1.59) |
| 51 | 0.28 | 0.60 | 1.10 (0.81,1.51) |

OR,odds ratio; CI,confidence interval
