# Supplementary material for: Risk factors and nomogram development for lymph node metastasis in early-onset early-stage gastric cancer: a retrospective cohort study
Source: Front Oncol. 2025 Apr 30;15:1544758. doi: 10.3389/fonc.2025.1544758 (PMC12074922; doi:10.3389/fonc.2025.1544758)
Supplement: Supplementary file 3 [file Table2.docx]

| **Characteristic** | **Patients No.(%)** | | | ***P* value** |
| --- | --- | --- | --- | --- |
|  | **Total** | **LMN- (n = 58)** | **LMN+(n = 18)** |  |
| Gender, n (%) |  |  |  | 0.543 |
| Female | 45 (59.2) | 33 (56.9) | 12 (66.7) |  |
| Male | 31 (40.8) | 25 (43.1) | 6 (33.3) |  |
| Age, Mean ± SD | 39.46 ± 4.71 | 39.57 ± 4.87 | 39.11 ± 4.25 | 0.721 |
| Location, n (%) |  |  |  | 0.097 |
| Gastric angle | 12 (15.8) | 12 (20.7) | 0 (0) |  |
| Gastric antrum | 33 (43.4) | 23 (39.7) | 10 (55.6) |  |
| Gastric corpus | 31 (40.8) | 23 (39.7) | 8 (44.4) |  |
| Degree of differentiation, n (%) |  |  |  | 0.133 |
| High | 4 ( 5.3) | 4 (6.9) | 0 (0) |  |
| Low | 58 (76.3) | 46 (79.3) | 12 (66.7) |  |
| Middle | 14 (18.4) | 8 (13.8) | 6 (33.3) |  |
| Pathological type, n (%) |  |  |  | 0.062 |
| Adenocarcinoma | 16 (21.1) | 15 (25.9) | 1 (5.6) |  |
| Adenocarcinoma with Signet-ring cell | 50 (65.8) | 34 (58.6) | 16 (88.9) |  |
| Signet-ring cell | 10 (13.2) | 9 (15.5) | 1 (5.6) |  |
| Invasion.depth, n (%) |  |  |  | < 0.001 |
| Mucosa | 52 (68.4) | 46 (79.3) | 6 (33.3) |  |
| Submucosa | 24 (31.6) | 12 (20.7) | 12 (66.7) |  |
| Lymphovascular space invasion, n (%) |  |  |  | 0.054 |
| No | 74 (97.4) | 58 (100) | 16 (88.9) |  |
| Yes | 2 ( 2.6) | 0 (0) | 2 (11.1) |  |
| Neural invasion, n (%) |  |  |  | 0.42 |
| No | 74 (97.4) | 57 (98.3) | 17 (94.4) |  |
| Yes | 2 ( 2.6) | 1 (1.7) | 1 (5.6) |  |
| Tumor maximum diameter, Median (IQR) | 1.5 (0.9, 2.0) | 1.4 (0.8, 2.0) | 2.0 (1.6, 2.9) | 0.002 |

**Table S2**. Characteristics of early onset early stage gastric cancer of external validation dataset with and without lymph node metastasis.

LNM+,positive lymph node metastase; LNM-, negative lymph node metastasis; SD,standard deviation; IQR, interquartile range
