# Supplementary material for: Risk factors and nomogram development for lymph node metastasis in early-onset early-stage gastric cancer: a retrospective cohort study
Source: Front Oncol. 2025 Apr 30;15:1544758. doi: 10.3389/fonc.2025.1544758 (PMC12074922; doi:10.3389/fonc.2025.1544758)
Supplement: Supplementary file 4 [file Table3.docx]

**Table S3**: CT scan detection of lymph node metastasis in early gastric cancer

|  |  | **LNM-** | **LNM+** |
| --- | --- | --- | --- |
| Enhanced CT,n(%) |  |  |  |
| Yes | 163(81.9%) | 132(81.5%) | 31(83.8%) |
| No | 36(18.1%) | 30(18.5%) | 6(16.2%) |
| Enhanced CT result,n(%) |  |  |  |
| LNM- | 157(96.3%) | 131(99.2%) | 26(83.9%) |
| LNM+ | 6(3.7%) | 1(0.8%) | 5(16.1%) |

LNM+,positive lymph node metastase; LNM-, negative lymph node metastasis
